# Evolution and Expression of the Immune System of a Facultatively Anadromous Salmonid

**DOI:** 10.3389/fimmu.2021.568729

**Published:** 2021-02-26

**Authors:** Thomas J. Colgan, Peter A. Moran, Louise C. Archer, Robert Wynne, Stephen A. Hutton, Philip McGinnity, Thomas E. Reed

**Affiliations:** ^1^ School of Biological, Earth and Environmental Sciences, University College Cork, Cork, Ireland; ^2^ Environmental Research Institute, University College Cork, Cork, Ireland; ^3^ Marine Institute, Newport, Ireland

**Keywords:** facultatively anadromous, immunity, gene expression, gene duplications, sexual dimorphism, salmonids

## Abstract

Vertebrates have evolved a complex immune system required for the identification of and coordinated response to harmful pathogens. Migratory species spend periods of their life-cycle in more than one environment, and their immune system consequently faces a greater diversity of pathogens residing in different environments. In facultatively anadromous salmonids, individuals may spend parts of their life-cycle in freshwater and marine environments. For species such as the brown trout *Salmo trutta*, sexes differ in their life-histories with females more likely to migrate to sea while males are more likely to stay and complete their life-cycle in their natal river. Salmonids have also undergone a lineage-specific whole genome duplication event, which may provide novel immune innovations but our current understanding of the differences in salmonid immune expression between the sexes is limited. We characterized the brown trout immune gene repertoire, identifying a number of canonical immune genes in non-salmonid teleosts to be duplicated in *S. trutta*, with genes involved in innate and adaptive immunity. Through genome-wide transcriptional profiling (“RNA-seq”) of male and female livers to investigate sex differences in gene expression amplitude and alternative splicing, we identified immune genes as being generally male-biased in expression. Our study provides important insights into the evolutionary consequences of whole genome duplication events on the salmonid immune gene repertoire and how the sexes differ in constitutive immune expression.

## Introduction

Species that migrate face a range of challenges. First, the physical act of migration can be metabolically and energetically demanding, resulting in trade-offs with other metabolically intensive physiological processes, such as immunity, when resources are limiting (1–3). Second, migratory species move through different environments and hence may be exposed to different pathogens and parasites (4, 5). For aquatic species that exhibit diadromy—the ability to move between marine and freshwater environments—an efficient immune system is required to cope with the challenges imposed by living in, and moving between, different osmotic environments with different pathogen and parasite communities.

In vertebrates, a sophisticated immune system has evolved that performs two vital functions: 1) the recognition and distinction of invasive pathogenic organisms from normal cells (“self”), and 2) coordinating an appropriate response through triggering pathways responsible for the synthesis of effector molecules that directly or indirectly reduce or remove the pathogenic threat (6, 7). Aside from detection of non-self-pathogenic organisms, the immune system also functions in the removal of abnormal cells and thus provides an important role in reducing the development and onset of disease.

Given the importance of the immune system in preventing infection and establishment of disease, there is strong selection pressure acting on immune genes. In response to these pressures, immune genes are generally fast evolving (8, 9). Additional innovations in immune potential can also arise through tandem duplication, retrotransposition, larger scale duplication of chromosomal regions or entire chromosomes, as well as whole genome duplication events (WGD). For example, two rounds of WGD events are suggested to have contributed to the genesis of the adaptive immune system in vertebrates (10). Indeed, WGD events produce duplicated copies of all genes, which selection can act on resulting in retention or removal of one or both copies. In terms of removal, as with general duplication events, gene loss can occur through reduced purifying selection resulting in functional divergence between the copies. Accumulation of deleterious mutations may eventually lead to one copy becoming non-functional (11). Gene loss may also be adaptive. For example, loss of gene function in a duplicated copy may be adaptive in response to environmental challenges (12, 13), including pathogens (14, 15). Alternatively, after duplication of a gene, functional divergence can occur whereby one copy evolves a slightly different or entirely novel function relative to the other copy. Functional divergence can result in subfunctionalization, whereby individual copies specialize on different components of the same function originally performed by the ancestral gene pre-duplication (i.e., “division of labor”), or neofunctionalization, whereby one copy may evolve a novel function (16).

As stated previously, the continuous expression and activation of immunity can be metabolically costly, resulting in trade-offs with fecundity and longevity (17). In particular, the sexes can differ in their levels of immune function with greater immunocompetence generally evident in females in comparison to males (18–23). Lower immunocompetence in males has been attributed in proximate terms to differences in circulating levels of hormones and their effects, such as androgen, and in ultimate terms to differences in life-history strategies, with males investing more in reproduction and associated secondary sexual characters while females invest in immune function and longevity (24). Moreover, in facultatively migratory species, differences are often evident between the sexes in the rate or timing of migration (25–28), which in turn has implications for exposure to pathogens and parasites and investment in immune defense. Differences in the level of immunity between the sexes can be detected at the transcriptional level (22, 29–31), whereby genes associated with the immune system may differ in their expression between the sexes. Approaches such as genome-wide transcriptomics (“RNA-seq”) are important tools for high-resolution detection and profiling of genes that differ in expression amplitude, as well as splicing, between the sexes. Such approaches have been applied to improve our understanding of genes underlying sexually dimorphic traits in a variety of taxa (32–35), including immunity (36).

An interesting study system for understanding the evolution and expression of the immune system and how it differs between the sexes are the salmonids, a group of culturally, economically and ecologically important teleost fish (37). The salmonids consist of approximately 70 species across 11 genera that have evolved flexible life-histories with a diversity of ecological adaptations that allow for migration to, and survival in, a range of freshwater and marine aquatic environments (38). There is a gradient from entirely non-anadromous species (which complete their entire life cycles in fresh water) through facultatively anadromous species to species that are almost entirely anadromous (39–44). Within facultatively anadromous species, rates of anadromy and other migratory tactics can vary between populations, and even among individuals within populations, particularly between the sexes with females more likely to undergo migration than males (42, 43, 45, 46).

Survival for prolonged periods in different aquatic environments, which contain different pathogenic threats, requires an immune system that can detect and respond to a diverse array of immune challenges. These factors likely placed strong selection pressures that shaped immune system evolution but additional immune novelty in salmonids may be the result of the salmonid-specific WGD event. The common ancestor of salmonids underwent an autotetraploidization event approximately 80–100 mya (47–49), which is believed to have contributed to genomic and phenotypic innovation as well as speciation within the salmonids (50). While the timing of the WGD event and first appearance of the anadromy during salmonid evolution are highly temporally detached, speciation rates were shown to be elevated within anadromous salmonids compared to non-anadromous salmonids with ecological factors, such as climate cooling, rather than the WGD suggested as the primary drivers of anadromy-linked diversification (47). The sequencing of the Atlantic salmon (*Salmo salar*) genome revealed that approximately 25% of the genome is undergoing delayed rediploidization, which is associated with major chromosomal rearrangements (48). Delayed rediploidization has been ongoing in parallel with speciation events, which has led to the proposal of ‘lineage-specific ohnolog resolution’ (LORe) as a mechanism to understand the impact of delayed rediploidization on the functional divergence of ohnologs across lineages that share a common ancestral WGD event (50). Under LORe, species divergence occurs before the rediploidization process is complete resulting in functional divergence of ohnologs independently within each lineage. The alternative model predicts rediploidization is completed prior to species diversification resulting in functional divergence of ohnologs within a shared ancestor (“Ancestral Ohnologue Resolution” or “AORe” model).

Due to shared selection pressures acting on ohnologs within a common ancestor, ohnologs that diverged in an ancestor are predicted to possess conserved functions across modern lineages (50). Recent genomic studies on salmonids have found evidence of relatively high rates of retention of duplicated genes arising from this most recent WGD (>50% of genes being found in functional ohnolog pairs), as well as evidence of neofunctionalization, whereby copies may diverge and are suggested to perform novel functions (48, 51, 52). Indeed, the evolutionary consequences of such events have served as impetus to examine functional divergence among ohnologous genes with putative immunological roles in salmonids (53–56). Despite these advances in our fundamental understanding of salmonid immunology, we understand less for species, such as the facultatively anadromous, brown trout (*Salmo trutta*). Recent declines in sea migration of *S. trutta* populations in Ireland and Scotland have raised concerns over the impact of disease and parasites, such as sea lice, on brown trout health and population performance (57–59). Given the enormous selection pressures exerted by parasites on their hosts, host defenses, including components of the host immune system, would be required to adapt to tolerate or resist so as to increase host survival and fitness (60). Therefore, increasing our understanding of brown trout immunity is warranted.

Here we had three main aims: Firstly, to characterize predicted immune genes found in the brown trout genome. For this, we used comparative genomic approaches to identify these genes in *S. trutta* based on homology with immune genes annotated in model organisms, such as zebrafish (*Danio rerio*), mouse (*Mus musculus*) and human (*Homo sapiens*). Given the overall enlarged gene repertoire in salmonids due to the salmonid-specific WGD event, as well as the strong selection pressures placed on immune genes by pathogens from both marine and freshwater environments, we would expect retention of most canonical immune genes, as well as the potential expansion of beneficial immune gene families. Secondly, we aimed to investigate evolutionary patterns of functional conservation and divergence, including gene loss in *S. trutta* immune ohnologs. Our final aim was to identify immune genes with sex-biased expression profiles. For this approach, we performed transcriptomic analyses on the liver, an important immunocompetent organ (61, 62), and quantified differences between males and females in gene expression amplitude, as well as alternative splicing, to identify molecular processes underlying sex differences in immune transcription and regulation.

## Materials and Methods

### Identification of Putative Brown Trout Immune Homologs

To identify genes in brown trout with putative immune function, we obtained gene lists for annotated immune genes in the zebrafish, *Danio rerio* [obtained from Zebrafish Information Network (ZFIN) database; (63)], as well as for the mouse, *Mus musculus* (obtained from Mouse Genome Informatics (MFI) database (64) and human, *Homo sapiens* [obtained from ImmPort (65)]. Using biomaRt [v. 2.45.8; (66)], we parsed the Ensembl BioMart database to identify “high confidence” orthologs found in the brown trout genome based on homology (phylogenetic protein trees), as well as conserved synteny (gene order conservation score and whole genome alignment scores). The threshold for classification of a brown trout gene as a ‘high confidence’ ortholog included: 1) a minimum gene conservation score of 50, which indicates the percentage of how many of the four closest neighbors of a gene match been orthologous pairs (i.e., at least two (50%) of neighboring genes match); 2) a minimum whole genome alignment score of 50; and 3) a minimum protein percentage identity of 50%. This approach identified 2,275 brown trout genes with homology to immune genes in three model organisms (**Supplemental Information Table 1**).

### Identification of Putative Immune Genes Across Salmonids

As a preliminary measure to understand immune gene repertoires across salmonids, we investigated the presence of homologues of putative brown trout immune genes in other salmonid species. To identify homology relationships, we first followed the approach outlined by Gillard et al. (51), and obtained protein sequences for the predicted proteomes from Ensembl [release 101; (67)] for twelve teleost fish, including the zebrafish (*Danio rerio*; GRCz11: GCA_000002035.4), three-spined stickleback (*Gasterosteus aculeatus*; BROAD S1), Japanese medaka (*Oryzias latipes*; ASM223467v1: GCA_002234715.1), Northern pike (*Esox Lucius*; Eluc_v4: GCA_004634155.1), Atlantic herring (*Clupea harengus*; Ch_v2.02: GCA_900700415.1), Atlantic cod (*Gadus morhua*; gadMor1), and guppy (*Poecilia reticulata*; GCA_000633615.2), and the salmonids, rainbow trout (*Oncorhynchus mykiss*; Omyk_1.0: GCA_002163495.1), Coho salmon (*Oncorhynchus kisutch*; Okis_V2: GCA_002021735.2), Chinook salmon (*Oncorhynchus tshawytscha*; Otsh_v1.0: GCA_002872995.1), Atlantic salmon (*Salmo salar*; ICSASG_v2: GCA_000233375.4), and brown trout (*Salmo trutta*; fSalTru1.1: GCA_901001165.1). From Ensembl, we also obtained two mammalian outgroups, human, *Homo sapiens* (GRCh38.p13: GCA_000001405.28), and mouse, *Mus musculus* (GRCm38.p6: GCA_000001635.8). For protein FASTA files downloaded from Ensembl, we extracted the longest protein isoform per gene per species using the OrthoFinder script “primary_transcripts.py” [v.2.3.11; (68, 69)]. We used OrthoFinder to assign groups of orthologs based on protein sequence similarity. Multiple sequence alignment was performed for protein sequences within each orthogroup using MAFFT. Maximum-likelihood trees were estimated using FastTree as implemented within OrthoFinder. OrthoFinder constructed a total of 47,752 orthogroups of which 22,101 were species-specific groups (**Supplemental Information Table 2**). The percentage of genes per species represented in the orthogroups was high (92%–97.5%). We parsed these orthogroups using the putative brown trout immune genes identified through the Ensembl-based analysis (n = 2,275 genes) and identified putative immune homologs present in 1,227 orthogroups (**Supplemental Information Table 3**). As a secondary measure to understand immune gene expansions and losses within the salmonids, we ran OrthoFinder using only the five salmonid proteomes, as well as Northern pike, the closest related species that did not undergo a fourth WGD event (70). While other salmonid genomes are publicly available, we restricted our analysis to Ensembl-generated datasets to account for gene models being predicted using a similar annotation pipeline (**Supplemental Information Table 4**). The presence of single copy orthologs in both Northern pike and all sequenced salmonids suggests that gene loss occurred in a common ancestor of modern salmonids soon after the WGD event or multiple independent losses have occurred across the salmonids.

### Expression of Brown Trout Immune Genes

To examine the functional expression of putative immune genes of *S. trutta*, we obtained available RNA-seq libraries for eight tissues from the NCBI (National Center for Biotechnology Information) Short Read Archive (SRA) database (BioProject: PRJEB33055). For each sample, we quantified transcript abundance using the quasialigner, Salmon [v.0.12.0; (71)]. Using these transcript abundances, we calculated gene-level counts using tximport [v.1.14.2; (72); **Supplemental Information Table 5**] and corrected for library-sizes using DESeq2 [v.1.26.0; (73)]. For each tissue, we quantified the total number of immune genes expressed per tissue, as well as compared relative abundance of immune gene expression for each tissue against non-immune genes.

### Identification of Putative Immune Ohnologs

To identify putative immune ohnologs, we first extracted within-species paralogs for brown trout using the Ensembl BioMart database (filter: “with_strutta_paralog”). Ensembl employs a pipeline that through protein trees can time and predict the last common ancestor for paralogs. We then subsetted putative immune genes identifying putative species-specific paralogs (n = 456), genus-specific (n = 344), as well as paralogs predicted to have arisen in the Salmoninae ancestor, which may represent paralogs generated as a result of the Salmonid-specific WGD. For each of these “Salmoninae” paralogs present in the brown trout genome, we subsetted and retained paralogs that shared at least 85% protein sequence similarity. We then obtained the Northern pike ortholog of each putative paralog. We extracted paralog pairs where only a single non-duplicated pike ortholog was evident, which matched only two paralogs in brown trout. For this, we kept only Northern pike orthologs that shared at least 85% sequence similarity to each of the brown trout paralogs. We also investigated the physical genomic coordinates of each ohnolog identifying that collinearity among putative immune ohnolog pairs is a global feature of the data, consistent with these genes being ohnologs. We therefore consider these brown trout paralogs as putative ohnologs. We found no significant difference (paired two sample t-test; *p* > 0.05) in predicted protein length between each ohnolog pair or their respective non-duplicated ortholog in Northern pike. This approach resulted in the identification of 434 ohnolog pairs (n = 868 genes) with potential immune function. We also extracted Atlantic salmon homologs of putative immune ohnolog pairs in brown trout and compared overlap with the *S. salar* ohnolog pairs described by Bertolotti et al. (74). Of the 434 brown trout immune ohnolog pairs, 408 pairs shared homologs in Atlantic salmon. Of this number, 88% (n = 362) were also identified as ohnolog pairs within the analysis of Bertolotti et al. providing independent evidence for the classification of such immune genes as ohnologs.

### Ohnolog Analysis of Immune Genes

#### Assessment of Putative Functional Conservation and Divergence

For each ohnolog pair, we estimated the evolutionary distance between each pair and the non-duplicated ortholog in Northern pike using distmat from EMBOSS [v.6.6.0; (75)] (in terms of amino acid substitutions per 100 amino acids; **Supplemental Information Table 6**). This allowed for the determination of copies that were more conserved or diverged in terms of protein sequence while accounting for variation in predicted amino acid length. To explore variation in functional domain architecture, we obtained InterPro functional domains assigned to predicted proteins for each pair from Ensembl BioMart (67). We then counted and compared the number of assigned domains for each ohnolog pair to identify any differences, which may be consistent with the genes performing different functions. As variation in protein length may explain variation in protein domains, we also compared predicted protein lengths between ohnologs identifying no significant difference in length (paired two sample t-test; *p* = 0.6).

#### Assessment of Variation in Expression Profiles Between Ohnologs

Using the gene-level counts generated by eight tissues, we generated co-expression clusters for putative immune ohnologs using Clust [v.1.10.8; (76)]. Here differences in expression profiles between ohnologs may reflect functional divergence, which has been determined in other salmonid species (48, 49, 51, 77). To determine divergence, we extracted ohnolog pairs that were assigned to different co-expression clusters and used corrected counts per sample generated by Clust for visualization purposes. Gene assignments to clusters are provided in **Supplemental Information Table 7**.

#### Assessment of Variation in Selection Patterns Acting on Ohnologs

To understand differences in selection acting on each pair, we calculated *dN/dS* ratios between each brown trout ohnolog and their respective non-duplicated Northern pike ortholog (**Supplemental Information Table 6**). We first aligned predicted protein sequences using clustal Omega [v.1.2.4; (78)] and converted the aligned sequences to nucleic acids using pal2nal [v.14; (79)] to obtain codon-based nucleic acid alignments. We then ran codeML as part of PAML [v.4.9; (80)] to estimate *dN/dS* ratios.

### Sampling of Laboratory-Based Brown Trout

To provide additional functional information on brown trout immune genes, and to examine potential sex differences in immune expression, we sampled livers from mature male and female *S. trutta* for transcriptomic analyses (**Supplemental Information Table 8**). For the present study, the fish used originated from a larger experimental aquaculture project that explored the expression of alternative life history tactics in brown trout (81, 82). This wider set-up involved 18 different tanks all connected within a recirculating aquaculture system (RAS), but in the current analysis, all our sampled fish came from two independent tanks (with each tank comprising fish from a different genetic background). Full details on the origins of the fish, fish husbandry procedures and other general information are given in Archer et al. (81, 82).

### Tissue Collection for Gene Expression Analysis

All fish were dissected between May and June 2018 (the endpoint of the larger experiment) when the fish were between two and three calendar years old. We collected livers from each individual fish and transferred the tissues to fresh 2 ml Eppendorf tubes containing RNALater solution. Samples were kept for 24 h at room temperature, and then stored at -80°C for later analysis. Samples during dissection were visually checked for mature testes or ovaries. For confirmation of genetic sex, we also obtained caudal fin clips from each fish during dissection and stored in 100% ethanol before subsequent genotyping.

### RNA Extraction, Purification, and Quality Assessment

Total RNA was extracted from a total of 37 tissues using TRIzol. Specifically, we extracted RNA from fifteen livers from males and from 22 livers from females. For RNA extractions, we removed each sample from -80°C long-term storage and incubated them on ice to thaw. Using a pipette, the RNALater solution was removed. We added 1ml of autoclaved phosphate-buffered saline to each tissue to briefly wash them before using sterilized forceps to transfer each washed tissue to an individual 2ml screw-cap homogenization tube. To each sample, 200ul of TRIzol was added and the sample was transferred to -80°C storage. Tissue disruption was performed using a 2 mm steel bead and a Tissuslyer II (Qiagen, UK). To each sample, a 2 mm steel bead was added and samples were homogenized at 30 Hz for 30 s. Post-homogenization, samples were visually inspected to ensure thorough disruption. Total RNA was extracted using chloroform followed by isopropanol precipitation. Precipitated RNA was washed using three washes of ethanol before elution in the elution buffer (Sigma, UK). Total RNA was purified using the Sigma GenElute Mammalian Total RNA kit. Quality assessment was initially performed using a NanoDrop ND-1000 (ThermoFisher, UK) while an accurate assessment of quantity was estimated using the Qubit fluorometer, followed by a TapeStation 2200 (Agilent, UK).

### Library Preparation and Sequencing

mRNA-enriched library preparation was performed for each individual sample using the NEBNext^®^ Ultra™ RNA Library Preparation kit and sequencing performed on an Illumina NovaSeq6000. Library preparation and sequencing was performed by NovoGene, Hong Kong. Sequencing resulted in a median of 26.1 million paired-end (PE) reads (2*150 bp) per individual (min. 20.1 million PE reads; max. 34.2 million PE reads). A combined total of 980 million PE reads were generated. Summary of sample information is provided in **Supplemental Information Table 8**.

### Quality Assessment of Raw Sequences

We quality assessed raw FASTQ sequences using FastQC [v.0.11.8; (83)] to identify adaptor contamination and sequences of low quality. Raw reads were aligned against the reference genome assembly (GCA_901001164.1) for *Salmo trutta* using STAR [v.2.7.0a; (84)]. Alignment statistics were calculated using samtools flagstat [v.1.9; (85)]. Summary statistics of alignments were compiled using Qualimap [v.2.2.1; (86)] and the output visualized using MultiQC [v.1.7; (87)].

### Transcript Abundance, Gene-Level Estimates, and Differential Expression Analysis

We quantified transcript abundance using two complementary approaches. Similar to the approach outlined above, using Salmon, we quasialigned raw reads against cDNA sequences for coding and non-coding genes available for *S. trutta* from Ensembl. We calculated gene-level counts using tximport and loaded these values into a DESeq2 object using DESeq2. Raw gene-level counts are provided in **Supplemental Information Table 9**. We performed a Wald test implemented by DESeq2 to identify significantly differentially expressed genes between males and females (Benjamini-Hochberg (BH) adjusted *p* < 0.05; **Supplemental Information Table 10**). To explore similarities in expression profiles across all samples, a principal component analysis was performed with DESeq2 for all samples using gene-level counts for 33,228 genes expressed in the liver following a variance stabilization transformation implemented by DESeq2. As a complementary measure, for each individual, we examined gene expression using a traditional aligner-based approach. We aligned reads against the brown trout reference genome assembly using STAR and extracted gene-level counts from the resultant BAM files using HTSeq [v. 0.11.2; (88); **Supplemental Information Table 11**]. We found an extremely high correlation (Pearson’s correlation: r=0.9, *p* < 2.2e^-16^) between the mean gene-level counts for both this approach, as well as the Salmon approach outlined above. This is also true for comparisons for individual samples across both approaches (lowest r=0.83, highest r=0.97). As an additional measure, we analyzed and compared the number of differentially expressed genes identified with DESeq2 when using the output of either transcript quantification approach (**Supplemental Information Table 12**). We found that >85% of genes identified as significantly differentially expressed between the sexes by Salmon were also identified as significant by the STAR-HTSeq approach. As quasi- and pseudoaligners, such as Salmon and kallisto, have higher accuracy and consistency in transcript quantification compared to traditional aligners (89), we report only the findings of our Salmon analysis.

### Differential Intron Usage Between the Sexes

While the sexes may express genes at different amplitudes, they may also express different isoforms of the same gene. We used the intron-splice analyzer leafcutter [v.0.2.8; (90)] to investigate differences between the sexes. Briefly, for each sample, we aligned raw reads against the brown trout genome assembly (GCA_901001165.1) using STAR (–outSAMstrandField intronMotif, –twopassMode Basic). We extracted splice junctions using bam2junc.sh and generated intron clusters using leafcutter.py (parameters: –minclureads 50, –maxintronlen 500000, –minreads 5). We then performed differential intron analysis using leafcutter_ds.R whereby for a cluster to be included it must be identified within at least five individuals within each sex (parameters: –min_samples_per_intron 5, –min_samples_per_group 5) to allow for investigation of genes with signatures of differential intron usage between the sexes. Leafcutter implements a log likelihood ratio test comparing a null model that assumes there is no difference between groups and an alternative model, which does. We adjusted resulting *p* values for multiple testing (False Discovery Rate < 0.05; **Supplemental Information Table 13**).

### Gene Ontology Term Enrichment Analysis of Immune and Sex-Biased Genes

Gene Ontology term enrichment analysis and visualization of outputs were performed using modified scripts generated by Colgan et al. (91). As the zebrafish is a model organism with extensive functional annotation of genes, we assigned GO terms for genes in the *Danio rerio* genome to putative *S. trutta* orthologs using biomaRt. We performed GO term enrichment analysis using topGO [v.2.34; (92)] with the ‘weight01’ algorithm and a node size of 20. The background universe consisted of all genes (n = 29,527) annotated with a Gene Ontology term.

**Figure 1:**
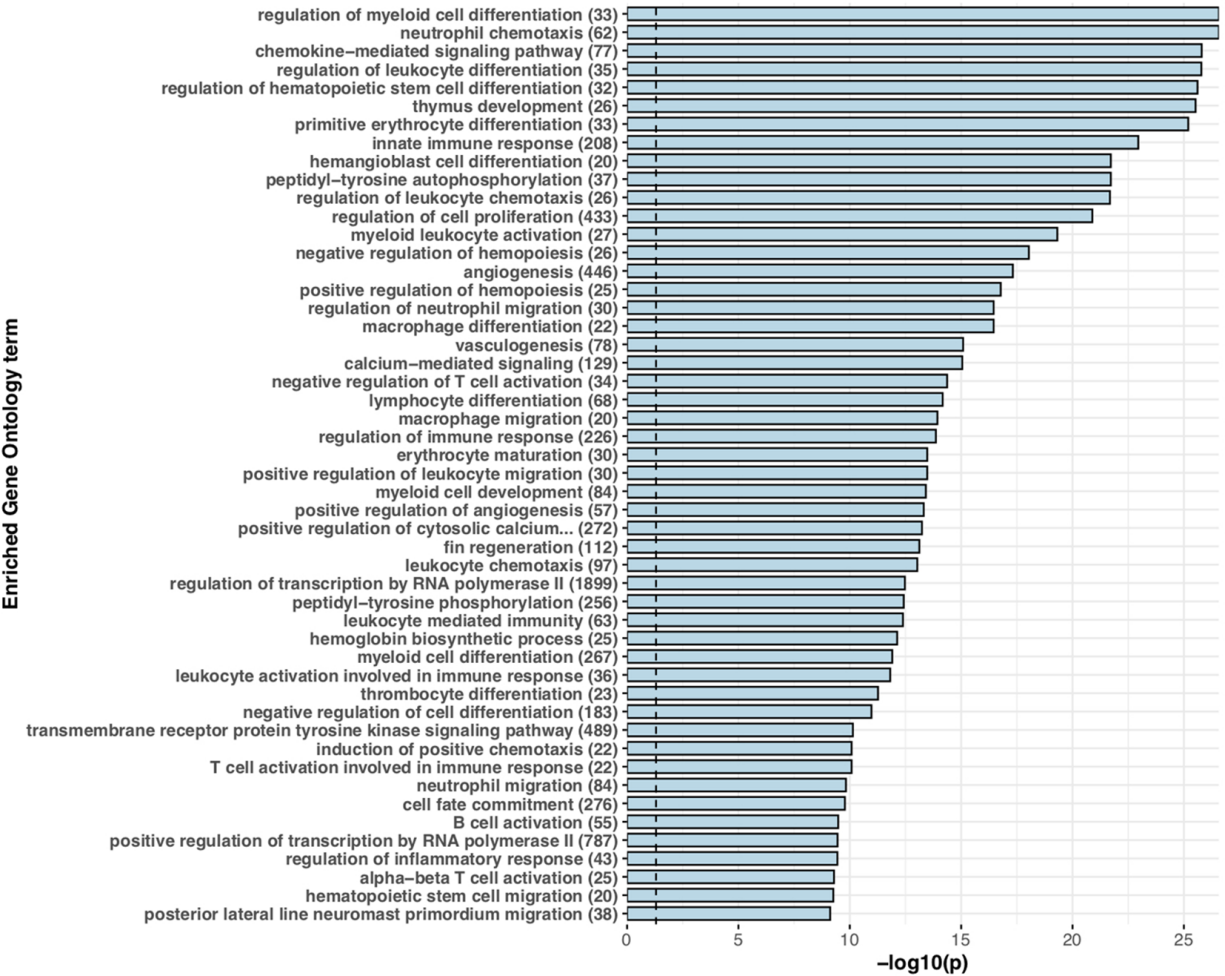
Expanded immune gene repertoire in the brown trout genome. Barchart displaying the top 50 significantly enriched ‘biological process’ associated Gene Ontology terms for putative immune genes with more than one copy in the brown trout genome in comparison to non-duplicated single copies in the Northern pike genome. For each significantly enriched term (Benjamini-Hochberg adjusted p < 0.05), the -log10 transformed p value is provided. For each term, the number of genes annotated with that specific term in the predicted *S. trutta* proteome is provided. The blue dotted vertical line represents threshold of significance (-log10 transformed p = 0.05).

For functional annotation of putative *S. trutta* immune genes, we performed a Fisher’s Exact test to identify enrichment of GO terms for species-specific duplications, genus-specific and putative immune ohnologs (**Figure 1**). For sex-biased immune genes, we performed Gene Ontology term enrichment analysis using a Kolmogorov-Smirnov (K-S) test. For all tests, we corrected for multiple testing using the Benjamini-Hochberg procedure and only reported terms as significant with an adjusted *p* < 0.05.

### Statistical Tests

For comparison between metrics associated with ohnologs, including variation in raw gene expression, predicted amino acid length, *dN/dS* ratios and evolutionary distance to non-duplicated ortholog outgroup, we used base statistical functions in R (v. 3.5.1). For pairwise comparison of means, we used Welch Two sample t-tests or Wilcoxon rank sum test. We tested for correlations using Pearson’s product moment correlation coefficient.

## Results

### Functional Annotation of *Salmo trutta* Immune Genes

**Figure 2:**
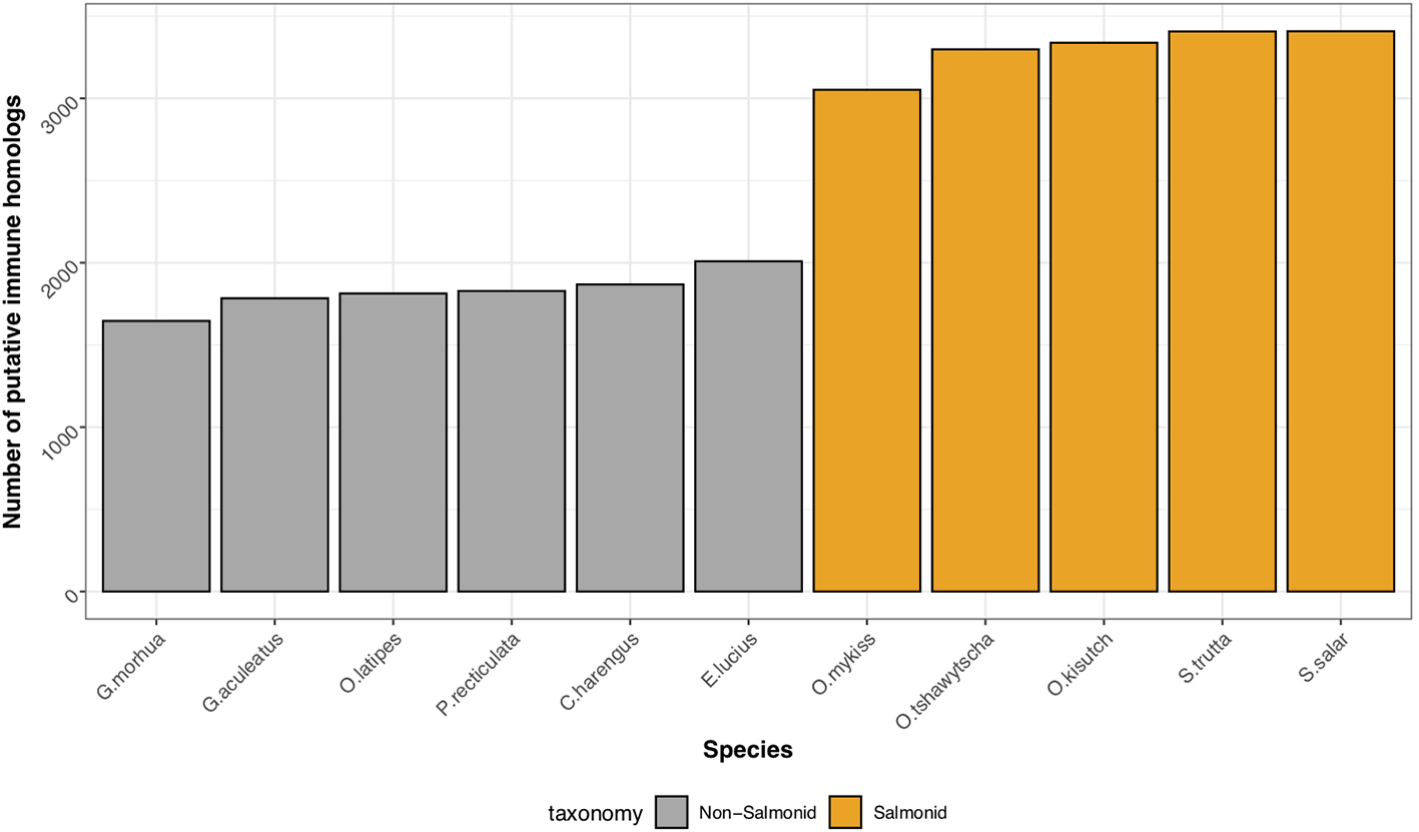
Conservation of immune gene repertoire in salmonid genomes. Histogram displaying the number of putative immune genes found within representative genomes of salmonid (Atlantic salmon, *S. salar*; Brown trout, *S. trutta*, Rainbow trout, *O. mykiss*, Coho salmon, *O. kisutch*, Chinook salmon, *O. tshawytscha*), and non-salmonid teleost fish (Northern pike, *E. lucius*; Atlantic herring, *C. harengus*; guppy, *P. reticulata*; Japanese medaka, *O. latipes*; three-spined stickleback, *G. aculeatus*; Atlantic cod, *G. morhua*).

Through comparison with annotated immune genes in zebrafish, mouse and human, we identified 2,275 putative homologs encoded by the brown trout genome (**Supplemental Information Table 1**). As expected, due to the salmonid-specific WGD event, the number of genes with putative roles in the immune system were elevated in salmonids in comparison to non-salmonid fishes (**Figure 2**) with the analysis by OrthoFinder indicating an additional 1,132 homologous sequences based on sequence similarity alone. However, as the immune orthologs identified by Ensembl are assigned based on additional synteny-based information, we consider the 2,275 of higher confidence and therefore, we based the rest of our analyses on these genes.

**Figure 3:**
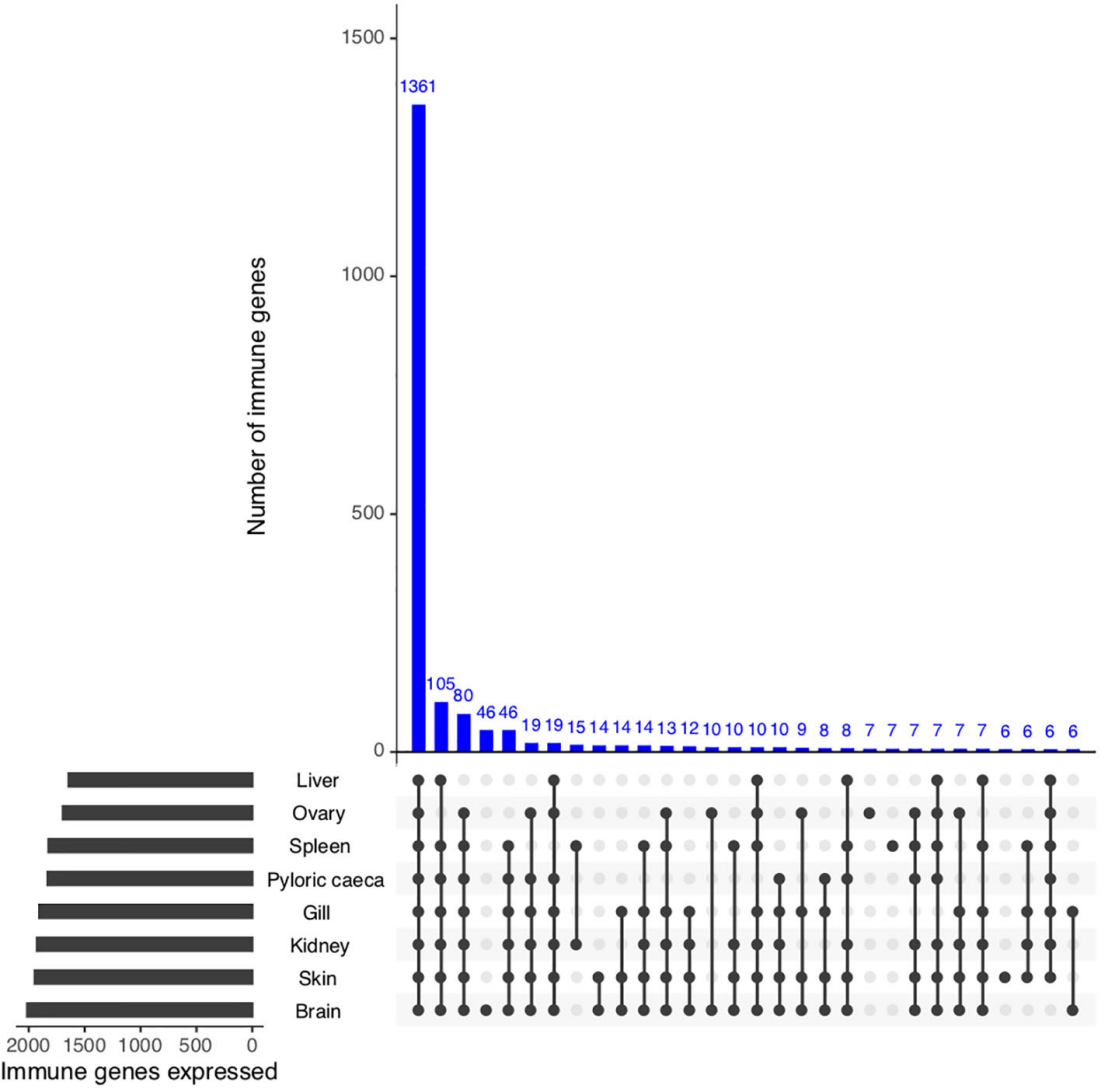
Functional validation of *S. trutta* immune expression across tissues. UpSet plot of immune gene expression across eight tissue types in brown trout. For each tissue, the plot contains a bar chart displaying the total number of immune genes expressed per tissue, as well as a histogram displaying the number of genes identified as expressed across tissues.

To identify if putative *S. trutta* immune genes are transcribed and therefore can be considered functional, we first investigated gene expression of all potential immune genes across eight available tissues (PRJEB33055). Collectively, we identified evidence of expression for 2,233 genes out of 2,275 (98.1% of immune genes) with 1,587 genes (69.8% of putative immune genes) expressed across all tissue types (**Figure 3**; **Supplemental Information Table 5**). We identified significantly higher expression (Wilcoxon test: *p* < 0.05) of immune genes in comparison to non-immune genes for all tissues examined, including immunocompetent organs, such as the spleen, kidney and liver, as well as the gills, which are an entry point for infection.

### Functional Conservation and Divergence in Immune Genes

To understand the evolutionary consequences for the immune system post salmonid-specific WGD, we compared the predicted brown trout gene complement to that of the Northern pike, *E. lucius*. Using the 2,275 putative immune genes in the brown trout genome, we obtained 2,000 ‘high-confidence’ orthologs in Northern pike. Of this number, 1,444 were present as 2:1 orthologs of Northern pike genes with 868 genes (434 pairs; **Supplemental Information Table 6**) annotated by Ensembl as paralogs with the time of duplication estimated within the Salmoninae, suggesting that these genes may be produced by the salmonid-specific WGD and represent ohnologs. Of the other 2:1 duplicate pairs, 30 (n = 60 genes) and 27 (n = 54 genes) were estimated to have duplicated within the genus *Salmo* and within *S. trutta*, respectively. The remainder may represent older duplication events, form part of expanded gene families in brown trout or represent gene losses in Northern pike. In terms of potential immune gene loss, we identified 253 immune genes with 1:1 copies in both *S. trutta* and Northern pike. Of these 253 genes, OrthoFinder also identified 127 as single copy across salmonids.

**Figure 4:**
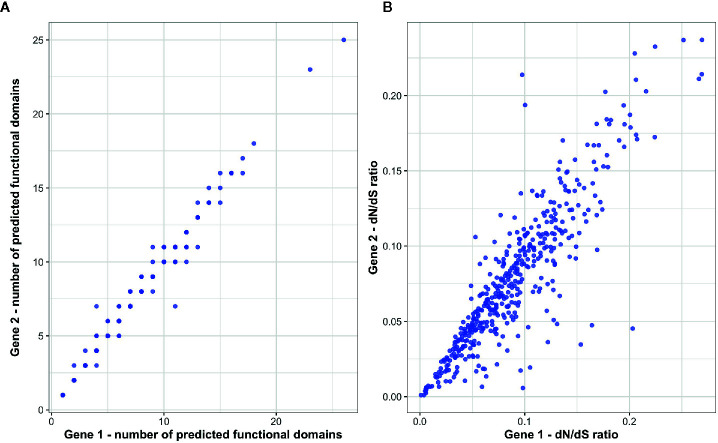
Assessment of functional divergence between *S. trutta* immune ohnolog pairs. **(A)** The number of predicted functional domains within the predicted protein coded for by each immune ohnolog. **(B)** For each ohnolog, *dN/dS* ratios were calculated for each and their respective non-duplicated ortholog in Northern pike.

To investigate patterns of functional conservation and divergence among immune ohnolog pairs, we first examined structural variation between ohnolog pairs in terms of the number of functional domains. Of the 434 immune ohnolog pairs, all proteins were annotated with at least one functional domain with 42 pairs differing in the number of functional domains with the more diverged copy generally having fewer domains suggestive that predicted proteins for at least ~11% of immune ohnolog have the potential to perform different functions. Overall, however, there was no significant difference in the number of predicted functional domains that each copy had (Wilcoxon test: *p >* 0.05; **Figure 4**). Similarly, there were no significant differences (Wilcoxon test: *p >* 0.05) in the mean gene, CDS or predicted protein length of ohnolog pairs. We also investigated variation between the pairs in terms of divergence from the single copy ortholog in Northern pike. Here, the more diverged copies based on evolutionary distance had significantly higher *dN/dS* ratios (paired two-sample t-test: *p* < 0.05; **Figure 4**) in comparison to more conserved copies but overall indicated that both copies for those pairs analyzed were under strong purifying selection (*dN/dS* < 0.25).

**Figure 5:**
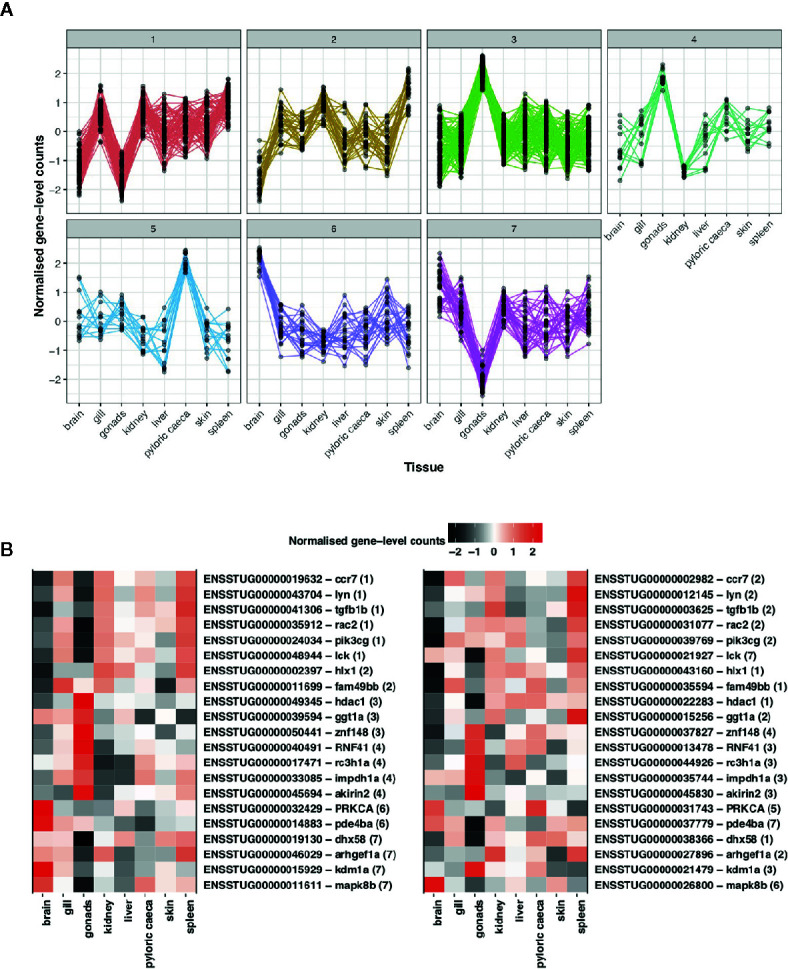
Assessment of divergence in *S. trutta* immune ohnolog expression profiles. **(A)** Clustering based on gene-level counts assigned 302 immune genes, which consist of one member of an ohnolog pair, to seven co-expression networks assigned based on the expression profile of each gene across eight tissue types. Each co-expression cluster is represented by a single line graph whereby the y-axis consists of gene-level counts normalized by Clust and the x-axis consists of eight tissues obtained from a single double haploid female. **(B)** Heatmap of expression profiles for ohnolog pairs where both ohnologs were assigned to different co-expression networks based on expression profiles across eight tissue types. Ensembl gene ID and description (separated by hyphen) are provided on the y-axis with number in parentheses indicating the cluster the gene was assigned to. The x-axis represents the eight tissues used to construct the co-expression networks.

To understand differences in gene expression profiles between immune ohnolog pairs, we performed a hierarchical clustering analysis to explore patterns of functional divergence. As a provisional measure, we first constructed co-expression networks using all putative immune genes identifying eight clusters consisting of 823 genes (smallest cluster = 20 genes; largest cluster = 257 genes). For immune ohnologs (n = 434 pairs), our analysis clustered 302 immune ohnologs into seven respective co-expression clusters (**Figure 5**). Of this number, 152 assigned genes belonged to ohnolog pairs (i.e., 76 pairs), where both ohnologs could be assigned to a co-expression network. The majority of these pairs (n = 55 pairs) were assigned to the same co-expression network indicating that both copies have conserved expression profiles across tissues suggestive that functions may also be conserved. Twenty one immune ohnolog pairs showed divergent co-expression profiles (**Figure 5**). For the remainder of the genes (n = 150) assigned to a cluster, they represent only one ohnolog from a pair where the other copy was unassigned due to lack of variation in expression or lack of expression across tissues.

### Assessment of Gene Loss in Brown Trout Immune Genes

Single copy genes present in salmonid genomes may be the result of adaptive loss or loss of a duplicated copy through neutral processes. First, using the reduced comparative dataset, we identified 4,297 single copy orthologs (SCOs) shared across the genomes of these six species. Of this number, 223 genes were annotated as putative immune genes but there was no evidence of significant enrichment of immune genes among all single copy orthologs $\left(X_{\text{df}=1}^{2}=0.54,p=0.46\right).$ We identified these genes as significantly enriched for the Gene Ontology term ‘toll-like receptor signaling pathway’ (GO:0002224). In total, these genes were identified as being significantly enriched (BH adjusted *p* < 0.05) for 26 terms with the most significant terms per GO category being ‘erythrocyte differentiation’, ‘immune response’, ‘complement activation’ and ‘cell chemotaxis’.

### Sexual Dimorphic Immune Expression Evident Within Brown Trout Liver

#### Differences in Gene Expression Amplitude Between the Sexes

**Figure 6:**
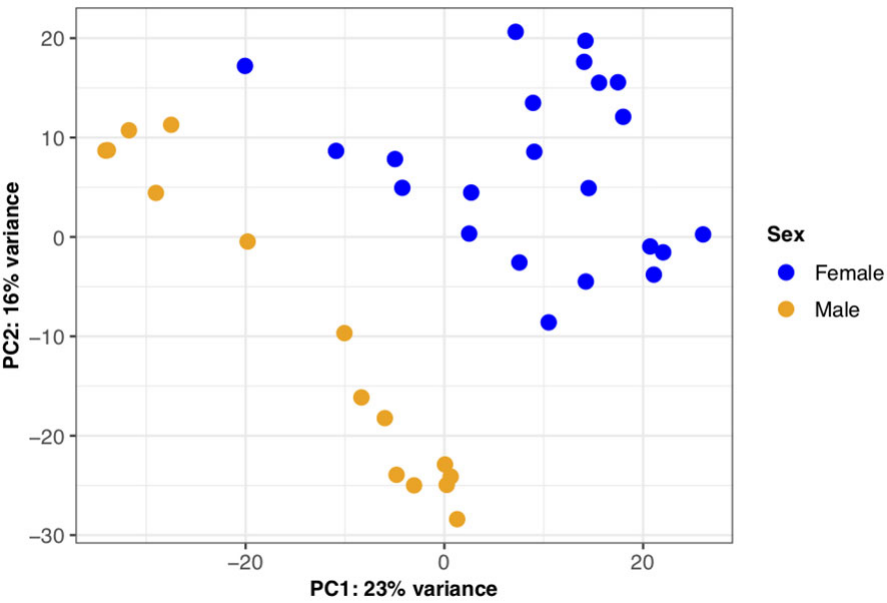
Sexually dimorphic gene expression in *S. trutta* liver. Principal component analysis for gene-level counts revealed sexually dimorphic gene expression in *S. trutta* liver. Principal component (PC1) explained 23% of variance with males and females clearly separating. Each dot on the scatterplot represent a single individual sample and are color-coded by sex (female = blue; male = orange).

To investigate overall differences in gene expression between the sexes, we first performed a principal component analysis. Principal component 1 (PC1) accounted for 23% of the variance in the dataset with PC1 largely separating males and females providing evidence of sex-biased expression profiles evident in the brown trout liver (**Figure 6**). We also find additional variation within PC2, which is likely attributed to differences in genetic background between tanks. Second, through differential expression analysis, we identified 3,689 genes as significantly differentially expressed (BH adjusted *p*‐value < 0.05) between the sexes (**Supplemental Information Table 10**). Of this number, the majority (n = 1,969) had a female-biased expression profile, which was a significant trend (binomial test, *p* < 10^-4^). Differentially expressed genes between the sexes were significantly enriched for 12 Gene Ontology terms with the most significant per GO category being “nucleobase catabolic process” (GO:0046113), “phosphatidylethanolamine binding” (GO:0008429) and “nucleolus” (GO:0005730) (**Supplemental Information Tables 14**-**16**).

**Figure 7:**
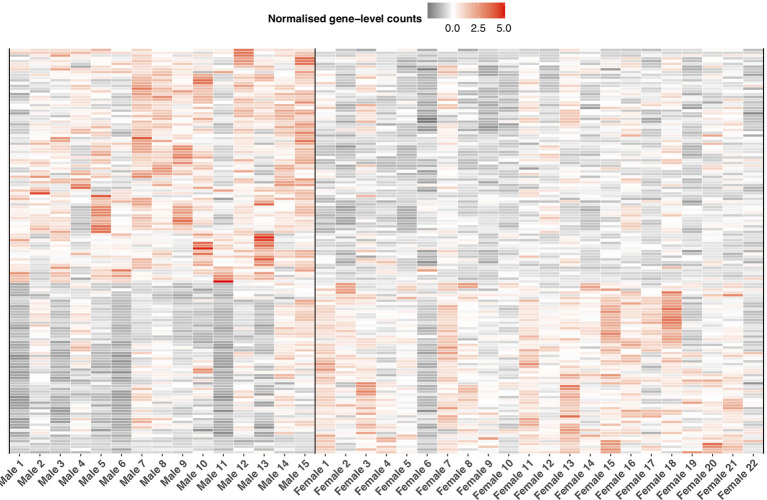
Sexually dimorphic immune expression in *S. trutta* liver. Heatmap displaying significantly differentially expressed immune genes between the sexes. Relative variation in immune gene expression is represented by a color gradient (gray = low, white = medium, red = high). Each column represents an individual trout with a vertical black line separating male from female samples. Each row represents expression level for an individual immune gene.

In relation to immune expression, we detected 83% of putative immune genes (n = 1,936 of 2,275) expressed in the liver. Of this number, we identified 269 genes as significantly differentially expressed (BH *p*< 0.05, **Figure 7**, **Supplementary Information Table 10**) between the sexes. Thirty-five of these 269 genes were annotated as single copy immune orthologs in the brown trout genome. In contrast to the entire transcriptome, which generally demonstrated female-biased expression, we detected more immune genes with higher gene expression in males (n = 158), which was more than expected by chance (binomial test, *p* < 0.03). Male-biased immune genes were significantly enriched (Fisher’s exact test; BH-adjusted *p* < 0.05) for 18 biological process-associated Gene Ontology terms, including ‘erythrocyte development’ (GO:0048821), ‘hemopoiesis’ (GO:0030097), and ‘response to cytokine’ (GO:0034097). For female-biased immune genes, we identified seven significantly enriched Gene Ontology terms associated with hemopoiesis and neutrophil differentiation.

#### Evidence of Alternative Splicing Between Sexes

We identified 218 intron clusters corresponding to 176 genes with evidence of significant alternative splicing (BH adjusted *p*‐value < 0.05; **Supplemental Information Table 13**) between males and females. Of this number, 55 were also significantly differentially expressed between the two sexes. We detected 15 alternatively spliced genes with roles in the salmonid immune system. Four of these genes were also differentially expressed between the sexes.

#### Conserved Sex-Biased Ohnolog Expression Among Immune Genes

We investigated if ohnologs demonstrated sex-biased gene expression and found 98 unique genes with sex-biased gene expression of which 12 ohnolog pairs exhibited significant differential expression for both genes (BH adjusted *p* < 0.05) between the sexes. For the majority (n = 10) of these ohnolog pairs, the ohnolog pairs have conserved differential expression whereby expression in both copies was elevated in the same sex compared to the other (n = 6 male-biased; n = 4 female-biased). Only for two ohnolog pairs did we identify differences in expression profiles between the ohnologs whereby the more conserved copies were all increased in males compared to females, but the less conserved copy had the opposite expression profile i.e., significantly reduced in males compared to females.

## Discussion

Migratory species, such as anadromous salmonids, require efficacious and adaptable immune systems to survive in a diversity of environments. Here we provide an important insight into the immune gene repertoire, as well as conservation and differences in gene expression across tissue types, for the facultatively anadromous brown trout *S. trutta*. Our findings indicate duplications and expansions of genes involved in immunological functions, such as chemotaxis and immune cell differentiation. Second, we assessed immune ohnologs for evidence of functional divergence in terms of domain architecture and gene expression profiles, identifying the majority to have conserved functional expression across tissue types in brown trout, while, surprisingly, only a few immune ohnolog pairs differed in terms of gene expression or the number of functional domains. We also identify evidence of immune gene loss in the salmonids. Lastly, we quantified sex-biased differences in immune gene expression in the brown trout liver, identifying the majority of differentially expressed immune genes to have male-biased expression. Our findings provide a novel insight into the immune complement of an ecologically and commercially important salmonid.

### Immune Gene Repertoire in Brown Trout: Retention and Expansion of Canonical Immune Genes

For teleost species that have undergone more recent whole genome duplication events, such as the salmonids, retention of duplicated genes may increase immune potential. In the brown trout genome, we identified high confidence orthologs for known canonical immune genes demonstrating that brown trout have essential genetic components of both innate and adaptive immune systems, as well as expansions (**Figures 1** and **2**). This number increases if we include protein homologs identified by OrthoFinder but additional synteny-based analyses would be required to confirm these genes as putative immune homologs. As our comparative analysis was mainly restricted to model species with well annotated immune repertoires, novel immune genes or genes highly diverged within the salmonids or restricted to *Salmo trutta* will not be reported. Gene expression analysis using eight available tissues indicates the vast majority of predicted immune genes as functional with expression evident across tissues (**Figure 3**). Retained duplicate copies were enriched for Gene Ontology terms associated with both innate and adaptive immunity. Among the most significantly enriched terms was neutrophil chemotaxis (**Figure 1**). Neutrophils are innate immune cells that are among the first responders to pathogen infection and inflammation and are a key aspect of the salmonid immune system. The response of these cells to chemical stimuli, known as chemotaxis, is important for the rapid response and subsequent migration of neutrophils to the site of signal origin (93). Neutrophils possess antimicrobial and phagocytic activity with the latter differing across teleost species (94). The expansion in brown trout of genes involved in chemotaxis may allow for the increased recognition of more diverse chemical stimulants, which may be beneficial for their migratory life-history that involves use of freshwater, brackish and marine environments.

Due to the complexity of the life-histories expressed by brown trout, immune repertoires are required for regulation and maintenance of immune expression during migration, an energetically stressful period, as well as to survive in both freshwater and marine environments, which can contain unique pathogenic threats (95). Variation in the strength of selection acting on duplicated genes can result in amino acid and/or regulatory divergence leading to the evolution of new functions or indeed, the process of pseudogenization and gene loss. Here, we found no significant difference in the overall number of functional domains between immune ohnologs in brown trout (**Figure 4**), as well as differences in expression profiles across tissues (**Figure 5**). However, for certain pairs, we did find evidence of domain architecture variation, as well as differences in expression profiles across tissues. We find variation in terms of functional domains for 42 pairs, and while future experimental validation is required, such pairs represent interesting candidates for investigating functional divergence in immune ohnologs. Similarly, 21 ohnolog pairs demonstrate divergence in gene expression profiles across eight tissues examined (**Figure 5**). As our analysis involved eight tissues rather than 15, which were previously used for Atlantic salmon (48) and rainbow trout (49), our power to detect divergent expression profiles may be reduced. Similarly, previous studies on salmonids explored conservation of ancestral function ohnologs through the construction of genome-wide co-expression networks, where one salmonid copy clustered by expression profile with that of a non-duplicated ortholog in the closely related, Northern pike, suggestive that one copy may retain and perform a conserved function across taxa. We did not explore such patterns in our analysis for brown trout due to the limited tissues available for both brown trout, of which only five were also available for Northern pike.

Of the ohnolog pairs with evidence of divergent gene expression profiles, such genes were annotated with a range of immunological function, including antiviral defense (*DHX58*, *zinc finger protein 148*), apoptosis (*RAC2*), inflammation regulation (*MAPK8*, *PDE4B, Roquin-1*), anti-microbial response (Akirin-2, *ARHGEF2*), T-cell activation (*LCK*, *HLX*) as well as tumor suppression (*PRKCA*, *FAM49B*, *IMPDH1*). Specific ohnolog pairs of interest included pairs where both genes were annotated as chemokine receptor type 7. In mammals, CC-chemokine receptor 7 (CCR7) is part of the G protein-coupled receptor family and can function in the activation of naive B and T lymphocytes with additional research suggesting the receptor may function in antiviral defense (96). In teleosts, the functions of chemokine receptors are less well understood but a CCR7 homolog has been previously characterized in the rainbow trout, *O. mykiss*, where based on sequence similarity, the predicted protein is suggested to perform a similar function to that of mammalian homologs (97, 98). The second ohnolog pair of interest were annotated as tyrosine-protein kinase Lyn. Lyn belongs to a Src- family of tyrosine kinases found in immune cells that can negatively regulate important signaling pathways (99). The gene is also a key mediator of pathways involved in B cell activation (99, 100) a function suggested as conserved in teleosts (101).

While extensive retention and functional divergence of ohnologs have been characterized in other salmonids, gene loss can also occur. Two primary molecular processes can lead to gene loss: as a consequence of an abrupt mutational event, such as an error in crossing over during meiosis, or through the slow accumulation of mutations during pseudogenization after an initial loss-of-function mutation (12). Comparative genomic studies have revealed biased patterns in gene loss in terms of functional bias with genes involved in certain cellular processes, such as DNA repair and transcription, more likely to be represented among genes where a copy has been lost (102, 103). In relation to WGD events, there is also evidence of genomic positional biases in terms of gene loss (104). Clusters of single copies may be due to reliance on similar transcriptional regulation machinery or architecture. Here through comparative analyses, we find gene loss conserved across salmonids. The conservation of synteny across salmonid genomes would suggest that loss of putative immune genes is non-random, as has been shown for other species (12). Future work will benefit from understanding the molecular, cellular and evolutionary consequences of gene loss in these species.

### Sexual Dimorphism in Trout Immune Expression

Sexes share largely the same genome but express it differently giving rise to different morphological and behavioral phenotypes. Here we investigated differences in gene expression in the liver, an organ previously used to understand salmonid metabolism gene expression (51), response to environmental stressors (105), as well as genes underlying sexual dimorphism (106). While the liver may have traditional roles in metabolism and antioxidant activity, a growing body of literature on mammalian and teleost immunology has provided important insights into the role of the liver in the innate immune response, immune tolerance and hematopoiesis (74, 107–112), as well as creating hostile molecular environments for parasites to migrate through (113, 114). Here we identified differential immune gene expression both in terms of expression amplitude (**Figures 6** **,** **7**) and splicing between the sexes. Immune genes exhibited a general male-bias in expression, which was in contrast to overall gene expression in the liver being female-biased. Immunological studies on sex differences in immune function in brown trout have suggested reduced immune function in mature males (115, 116), and therefore, females would have been expected to have higher immune gene expression compared to males. While our fish were laboratory reared and not directly immune challenged, they were maintained in normal lab environments (i.e., clean but not sterile) and therefore, we would expect a background level of immune gene expression. Sex-biased differences in expression could be due to anticipation of immune challenge. As male brown trout are less likely to undergo sea migration, remaining resident and completing their life-cycle in freshwater environments, pathogens present in freshwater environments are more likely to encounter males than females and therefore, parasite-mediated selection in brown trout may result in variation in environment-dependent immune expression between sexes. Indeed, males do suffer more severe infestations by freshwater ectoparasites in comparison to females (117), yet our understanding of immune potential and function is lacking.

An interesting finding among the genes with sex-biased differences was the presence of putative single copy orthologs. As sexes largely share the same genome but have different fitness optima and may express some genes differently, this can result in sexually antagonistic loci, which increase fitness when expressed in one sex but are detrimental in the other. Sex-biased gene expression has been suggested as a mechanism to resolve such conflict (118, 119). Aside from transcriptional regulation, modifications in genomic architecture, such as sex-dependent dominance (120), maintenance of sexually antagonistic loci on sex chromosomes (121) or duplication events (122, 123) may also resolve conflict. It is interesting therefore that since the salmonid WGD, duplicate copies of immune genes have been lost either through adaptive or neutral processes that may now be sexually antagonistic. The application of population genetic approaches, in combination with sex-biased gene expression, have been used to reveal genomic signatures of loci associated with sexual conflict (118, 124), which could be applied to future studies in brown trout and other salmonids to provide important insights into the evolutionary processes shaping sex differences.

## Conclusion

Salmonid genomics is a rapidly advancing field and is providing comprehensive insights into genes underlying phenotypically plastic traits, such as sea age at maturity, as well as genomic structures resolving sexually antagonistic loci (28, 120). Here we explore the consequences of a salmonid-specific WGD on immune gene repertoire in brown trout, finding that brown trout has an enlarged immune gene complement relative to non-salmonids, with many immune ohnologs retained that have conserved immune expression profiles between the pairs. We find preliminary signatures of some ohnologs coding for proteins that may have potential divergent functions between the pairs, but functional validation is required to determine the exact role these genes may play in the brown trout immune system. Lastly, we add to a growing body of research that explores key physiological differences among the sexes through the identification of differences in immune expression.

Our findings provide important insights into immune gene evolution and expression in a culturally, economically and ecologically important species. Like many species, brown trout face an uncertain future due to changing climates with increasing temperatures potentially leading to reduced sea migration rates (82) as well as potentially impacting immune function (125) while pathogens, such as sea lice, associated with increasing aquaculture, are also suggested to contribute to migratory declines (126). Improved understanding of immune potential, expression and function may benefit management strategies and conservation schemes for wild populations to assist in the maintenance of at-risk facultatively anadromous populations.

## Data Availability Statement

Raw sequence data files are deposited in the NCBI short read archive (BioProject ID: PRJNA667168). Scripts underpinning the data analysis are archived on GitHub (https://github.com/Joscolgan/salmonid_immune_study). Raw read counts used in the present analyses are provided as tables in **Supplementary Information**.

## Ethics Statement

The animal study was reviewed and approved by This study was carried out in accordance with the recommendations of the Health Products Regulatory Authority (HPRA) Ireland, under HPRA project license AE19130/P034, and HPRA individual licenses AE19130/I087, AE19130/I200, AE19130/I201, and AE19130/I202.

## Author Contributions

TC, PAM, PMcG, and TR designed the experiment. LA, RW, and SH harvested the liver samples. TC performed the RNA extractions and quality checks. TC performed the majority of analyses. All authors contributed to the article and approved the submitted version.

## Funding

This research was supported by an ERC Starting Grant (639192-ALH) and an SFI ERC Support Award awarded to TR. PMcG was funded by an SFI-DEL grant (2015 15/IA/3028) and the Marine Research Programme 2014–2020 RESPI/FS/16/01 (Marine Institute).

## Conflict of Interest

The authors declare that the research was conducted in the absence of any commercial or financial relationships that could be construed as a potential conflict of interest.
